# Supplementary material for: Iterative improvement in the automatic modular design of robot swarms
Source: PeerJ Comput Sci. 2020 Dec 7;6:e322. doi: 10.7717/peerj-cs.322 (PMC7924708; doi:10.7717/peerj-cs.322)
Supplement: Supplemental Information 3 [file peerj-cs-06-322-s003.zip › argos3/doc/api/standalone/a00541_source.html]

ARGoS: plugins/robots/generic/simulator/quadrotor\_speed\_default\_actuator.h Source File


- Main Page
- Related Pages
- Namespaces
- Classes
- Files

- File List
- File Members

# plugins/robots/generic/simulator/quadrotor\_speed\_default\_actuator.h

Go to the documentation of this file.

```
00001 
00007 #ifndef QUADROTOR_SPEED_ACTUATOR_DEFAULT_H
00008 #define QUADROTOR_SPEED_ACTUATOR_DEFAULT_H
00009 
00010 #include <string>
00011 #include <map>
00012 
00013 namespace argos {
00014    class CQuadRotorSpeedDefaultActuator;
00015 }
00016 
00017 #include <argos3/core/utility/math/rng.h>
00018 #include <argos3/core/simulator/actuator.h>
00019 #include <argos3/core/simulator/entity/composable_entity.h>
00020 #include <argos3/plugins/robots/generic/control_interface/ci_quadrotor_speed_actuator.h>
00021 #include <argos3/plugins/simulator/entities/quadrotor_entity.h>
00022 
00023 namespace argos {
00024 
00025    class CQuadRotorSpeedDefaultActuator : public CSimulatedActuator,
00026                                           public CCI_QuadRotorSpeedActuator {
00027 
00028    public:
00029 
00030       CQuadRotorSpeedDefaultActuator();
00031       virtual ~CQuadRotorSpeedDefaultActuator() {}
00032 
00033       virtual void SetRobot(CComposableEntity& c_entity);
00034 
00035       virtual void Init(TConfigurationNode& t_tree);
00036 
00037       virtual void SetLinearVelocity(const CVector3& c_velocity);
00038       virtual void SetRotationalSpeed(const CRadians& c_speed);
00039 
00040       virtual void Update();
00041       virtual void Reset();
00042 
00043    protected:
00044 
00045       CQuadRotorEntity* m_pcQuadRotorEntity;
00046       CEmbodiedEntity* m_pcEmbodiedEntity;
00047       CQuadRotorEntity::SSpeedControlData m_sDesiredSpeedData;
00048       
00049    };
00050 
00051 }
00052 
00053 #endif
```

---

Generated on 10 Jul 2018 for ARGoS by 
 1.6.1
